# The establishment of transient expression systems and their application for gene function analysis of flavonoid biosynthesis in *Carthamus tinctorius* L

**DOI:** 10.1186/s12870-023-04210-1

**Published:** 2023-04-10

**Authors:** Bin Xian, Ziqing Xi, Chaoxiang Ren, Jie Yan, Jiang Chen, Jin Pei

**Affiliations:** 1 State Key Laboratory of Southwestern Chinese Medicine Resources, Chengdu University of Traditional Chinese Medicine, Chengdu, 611137 Sichuan China; 2 College of Pharmacy, Chengdu University of Traditional Chinese Medicine, Chengdu, 611137 Sichuan China; 3 The State Bank of Chinese Drug Germplasm Resources, Chengdu University of Traditional Chinese Medicine, Chengdu, 611137 China

**Keywords:** Safflower callus, Transient expression system, Agrobacterium, Biolistic, Gene function analysis

## Abstract

**Background:**

Safflower (*Carthamus tinctorius* L.) is an important economic crop and a traditional medicinal material rich in flavonoids, which can alleviate cardiovascular and cerebrovascular pathologies. Thus, many candidate genes involved in safflower flavonoid biosynthesis have been cloned. However, owing to the lack of a homologous gene expression system, research on gene function is limited to model plants. Therefore, a gene function identification protocol for safflower must be established.

**Results:**

In the present study, using safflower callus as the experimental material, *Agrobacterium* and biolistic transient expression systems were established. In the *Agrobacterium* transient expression system, the highest transformation rate was obtained at the original *Agrobacterium* concentration of OD_600_ 0.4, infiltration concentration of OD_600_ 0.6, infection for 20 min, co-culture for 3 days, and acetosyringone concentration of 100 μmol·L^−1^. In the biolistic transient expression system, the highest transformation efficiency was observed at helium pressure of 1,350 psi, vacuum degree of -0.8 bar, flight distance of 6.5 cm, one round of bombardment, plasmid concentration of 3 μg·shot^−1^, and gold particle concentration of 100 μg·shot^−1^. Further, these two transient expression systems were used for the functional analysis of *CtCHS1* as an example. After overexpression, relative *CtCHS1* expression increased, particularly in *Agrobacterium*-transformed calli. Additionally, the contents of some flavonoids were altered; for instance, naringenin and genistein levels were significantly increased in *Agrobacterium-*transformed calli, whereas luteolin, luteolin-7-O-rutinoside, and apigenin derivative levels were significantly decreased in biolistic*-*transformed calli.

**Conclusion:**

Using safflower callus as the experimental material, highly efficient *Agrobacterium* and biolistic transient expression systems were successfully established, and the utility of both systems for investigating gene function was demonstrated. The proposed safflower callus transient expression systems will be useful for further functional analyses of flavonoid biosynthetic genes in safflower.

**Supplementary Information:**

The online version contains supplementary material available at 10.1186/s12870-023-04210-1.

## Background

Safflower (*Carthamus tinctorius* L.) is an annual herb belonging to the plant family Asteraceae. This plant is cultivated in many countries because of its great medicinal value and utility as an oil crop. Safflower flowers are rich in flavonoids, specifically hydroxysafflor yellow A (HSYA), which possesses antithrombotic, anti-inflammatory, and anti-oxidant properties; furthermore, flavonoids can protect ischemic and/or hypoxic cardiomyocytes and brain cells and have been widely used for the treatment of stroke, myocardial infarction, and other cardiovascular and/or cerebrovascular diseases [1, 2]. Owing to their significant medicinal value, the biosynthetic pathways of flavonoids in safflower have attracted much attention, and many genes involved in flavonoid synthesis have been cloned. However, owing to the lack of a verification system, functions of most genes remain unknown. Meanwhile, functions of some genes have been identified through heterologous expression in *Arabidopsis*. For instance, safflower chalcone isomerase (CHI) [3], cytochrome P450 (CYP) [4], and cysteine protease 1 (CP1) [5] have been functionally characterized using *Arabidopsis* models*.* However, many medicinal plants, such as safflower, contain unique secondary metabolites. In addition, *Arabidopsis* and safflower express significantly different evolutionary pathways and genetic backgrounds from Cruciferae to Compositae and from Archichlamydeae to Gamopetalae, resulting in the misinterpretation or incomplete recognition of gene function. Therefore, an effective homologous expression system for safflower must be established.

Transient expression systems have been widely used for elucidating gene functions in plants. Transient expression offers the advantages of being less affected by gene position and silencing effects, lack of heritable offspring for high biosafety, stable and high expression efficiency, simple operation, and short duration [6]. Calli are a material used for transient expression, because their loose texture is conducive to high transformation efficiency and they possess potential for further development into complete plants. *Agrobacterium* and biolistics are commonly used transient gene expression systems. *Agrobacterium*-mediated transformation offers the advantages of high gene transfer efficiency, feasibility of carrying longer gene fragments, and ease of RNA and protein analysis [7]. Meanwhile, biolistic-mediated transformation offers the advantage of feasibility to transform various plant tissues without host limitation [6]. The efficiency of *Agrobacterium*-mediated transient expression is affected by concentration, infiltration time, and acetosyringone (AS) concentration, whereas the efficiency of biolistic-mediated transient expression is affected by gold particle concentration, bombardment distance, and helium pressure [6]. In recent years, transient expression systems have ceased to be exclusive to model plants, such as *Arabidopsis* [8] and *Nicotiana benthamiana* [9], and individual transient expression systems for many economically important plants, such as cotton [10], *Ricinus* [11], citrus [12], *Chenopodium quinoa* [13], and *Vigna unguiculata* [14], have been established. However, compared to these economic crops, the establishment of transient expression systems in medicinal plants are very much behind, which may leave the functions of genes incomplete and hinders the development and utilization of these natural medicinal values by humans. Since safflower is an important medicinal plant with great potential for the treatment of cardiovascular diseases, it is essential to establish a transient expression system for the analysis of flavonoid synthesis-related gene functions.

In the present study, cotyledons obtained from sterile seedlings were used as explants to induce callus formation. Then, optimal conditions for the transient transformation of safflower calli mediated by *Agrobacterium* and biolistics were screened. Finally, the chalcone synthase gene (*CtCHS1*) [15] was overexpressed in safflower calli using the two transient expression systems to evaluate their efficiency. This work lays the foundation for further analyses of safflower-specific flavonoid biosynthetic pathways and offers insights for functional analyses of other genes in safflower.

## Results

### Callus induction in safflower

**Fig. 1:**
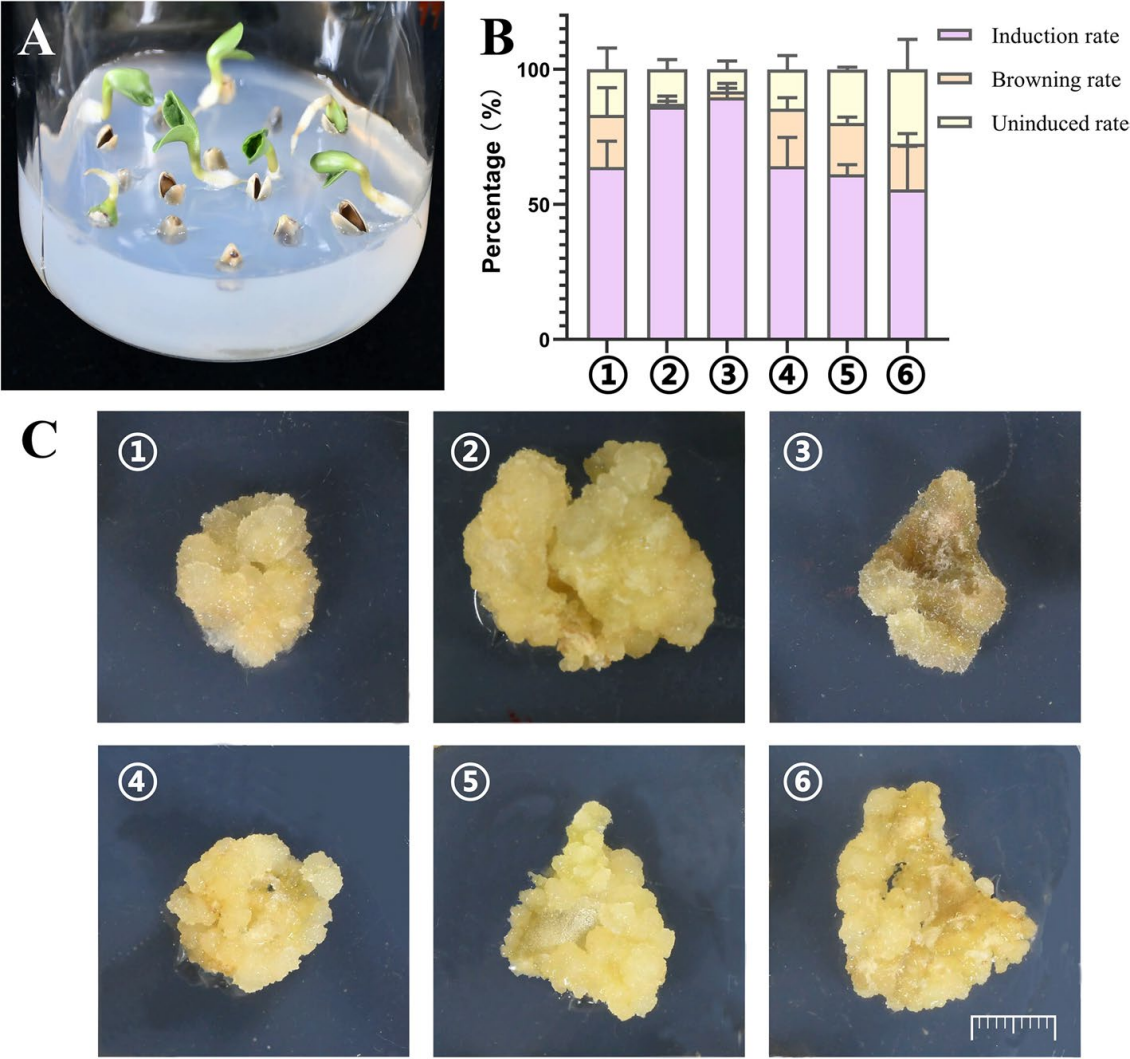
Callus induction of safflower. **A** The sterile seedlings of safflower. **B** Callus induction rate of different groups. **C** Safflower callus induced by 6 kinds of media

Sterilized mature safflower seeds were germinated in MS medium under a 16/8 h light/dark conditions at 25 ± 2℃. After 7 days of growth, sterile seedlings were obtained (Fig. 1A). The cotyledons of sterile seedlings were used as explants, which were inoculated in a medium containing different hormones to induce callus formation. After culturing for 40 day under dark conditions at 25 ± 2℃, many calli developed on the cotyledons. The calli with the light yellow, loose structure were grown in medium (2) (0.1 mg·L^−1^ NAA, 2 mg·L^−1^ 6-BA, and 15 mg·L^−1^ KT-30) (Fig. 1B); a high callus induction rate was noted in this medium as well (Fig. 1C).

### Agrobacterium-mediated transient expression in safflower callus

**Fig. 2:**
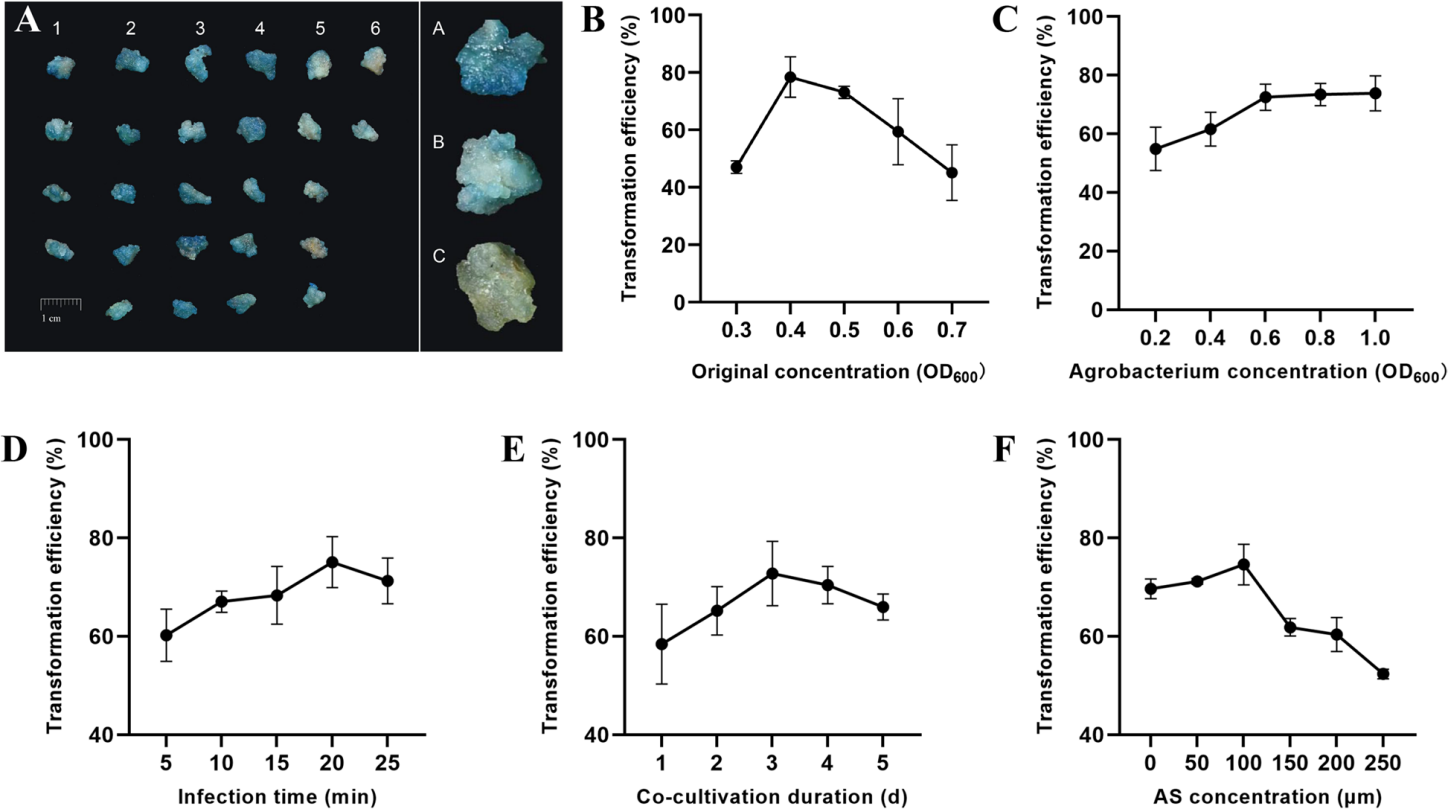
GUS expression analysis of safflower callus transformed by *Agrobacterium.* A GUS expression analysis of *Agrobacterium*-transformed safflower callus. 1, 2, 3, and 4 are considered as positive, and 5 and 6 are considered as negative; in the stained callus, A is positive, B is false positive, and C is negative control. B Effect of original concentration of *Agrobacterium* on transient transformation (OD600 values of 0.3, 0.4, 0.5, 0.6, and 0.7). C Effect of *Agrobacterium* concentration on transient transformation (OD600 values of 0.2, 0.4, 0.6, 0.8, and 1.0). D Effect of infection time on transient transformation (5, 10, 15, 20 and 25 min). E Effect of co-cultivation duration on transient transformation (1, 2, 3, 4 and 5 days). F Effect of AS concentration on transient transformation (50, 100, 150, 200 and 250 μm·L^−1^)

We investigated the effects of *Agrobacterium* original concentration, concentration, treatment time, co-cultivation time, and AS concentration on the transient transformation efficiency of safflower calli using single-factor experiments (Fig. 2). The highest transformation efficiency was obtained when the original concentration of *Agrobacterium* was at OD_600_ of 0.4, reaching 79.54% (Fig. 2B). When treated with different concentrations of *Agrobacterium*, the highest transformation efficiency of 72.52% (Fig. 2C) was recorded at the concentration of OD_600_ = 0.6. With increase in infection time, the transformation rate showed a gradual increasing trend; the highest transformation efficiency of 75.09% was obtained at 20 min (Fig. 2D), and efficiency decreased when the time exceeded 20 min. Co-cultivation for 3 days after infection resulted in the highest number of positive calli (72.76%) (Fig. 2E). However, with further increase in co-cultivation time, excessive propagation of *Agrobacterium* led to callus death, indicating a decline in transformation efficiency. AS promotes the expression of virulence genes of *Agrobacterium* and the transfer of the T-DNA (transfer DNA) region, which significantly improves transformation efficiency; the highest efficiency was observed at the AS concentration of 100 μmol·L^−1^ (Fig. 2F). However, excess AS concentration leads to cell death in plants; accordingly, a significant decrease in transformation efficiency was observed at the AS concentration of 250 μmol·L^−1^.

### Biolistic-mediated transient expression in safflower calli

**Fig. 3:**
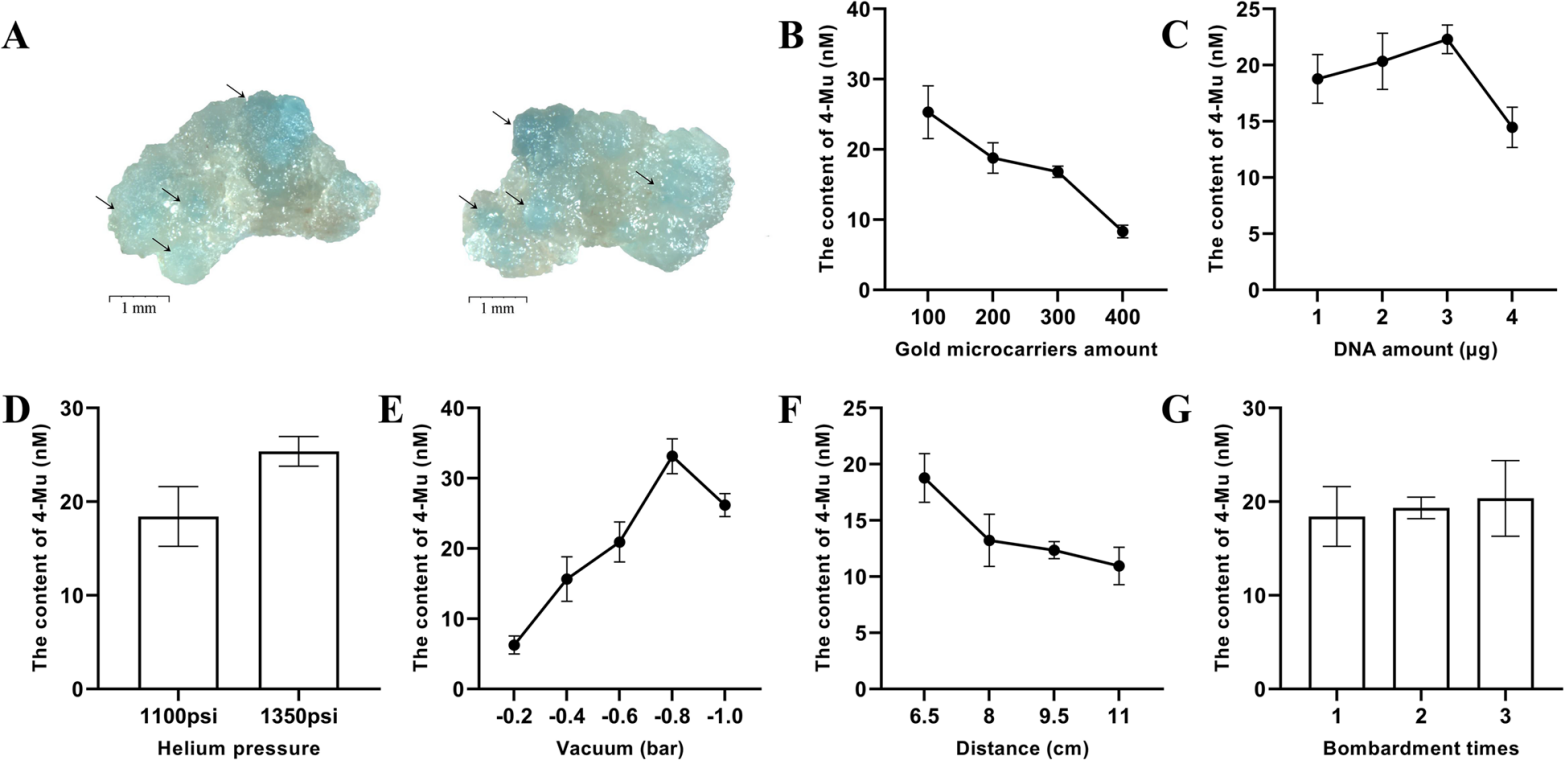
GUS enzyme activity assay of safflower callus transformed by biolistic. **A** GUS expression analysis of biolistic-transformed safflower callus. **B** Effect of gold particles amount on transient efficiency (100, 200, 300 and 400 μg·shot^−1^). **C** Effect of plasmids amount on transient efficiency (1, 2, 3 and μg·shot^−1^). **D** Effect of helium pressure on transient efficiency (1100 and 1350 psi). **E** Effect of vacuum on transient efficiency (-0.2, -0.4, -0.6, -0.8 and -1.0 bar). **F** Effect of flight distance on transient efficiency (6.5, 8, 9.5 and 11 cm). **G** Effect of bombardment times on transient efficiency (1, 2 and 3 times)

Optimal conditions to achieve the highest biolistic-mediated transient transformation efficiency of safflower callus (represented by the content of 4-methylumbelliferone) were explored. Six parameters, including gold particle concentration, plasmid concentration, helium pressure, vacuum, flight distance, and number of bombardments, were evaluated (Fig. 3). In all experiments, the highest transformation efficiency was obtained at the vacuum of -0.8 bar, with the 4-MU (4-methylumbelliferone) level of 33.13 nM (Fig. 3E). In helium pressure experiments, the efficiency was higher at 1,350 psi (Fig. 3D). When transforming calli at different flight distances, the highest transformation efficiency was obtained at a distance of 6.5 cm (Fig. 3F). Increasing plasmid concentration increased *β*-glucuronidase (GUS) expression; as such, when plasmid concentration was 3 ng·shot^−1^, the highest level of 4-MU was detected (22.31 nM) (Fig. 3C). However, with further increase in plasmid concentration, gold particle dispersion was reduced, which decreased transformation efficiency. The callus transformation efficiency decreased with increase in gold particle concentration. As such, 4-MU level decreased from 25.30 to 8.32 nM with increase in gold particle concentration from 100 to 400 μg·shot^−1^ (Fig. 3B). However, the number of bombardments did not significantly affect the efficiency of GUS expression (Fig. 3G).

### Agrobacterium- and biolistic-mediated overexpression of CtCHS1 in safflower calli

**Fig. 4:**
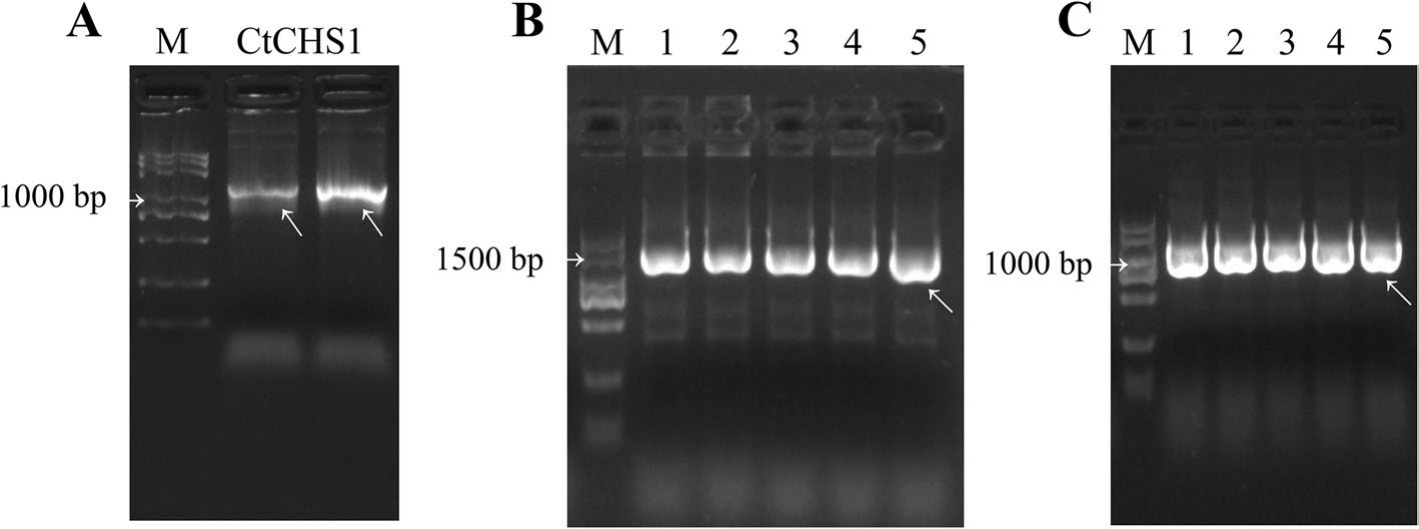
*CtCHS1* gene cloning and vector construction. **A** PCR amplification results of *CtCHS1* gene. **B** Vector construction of *pRI201- CtCHS1*. **C** Vector construction of *pBI221- CtCHS1*

**Fig. 5:**
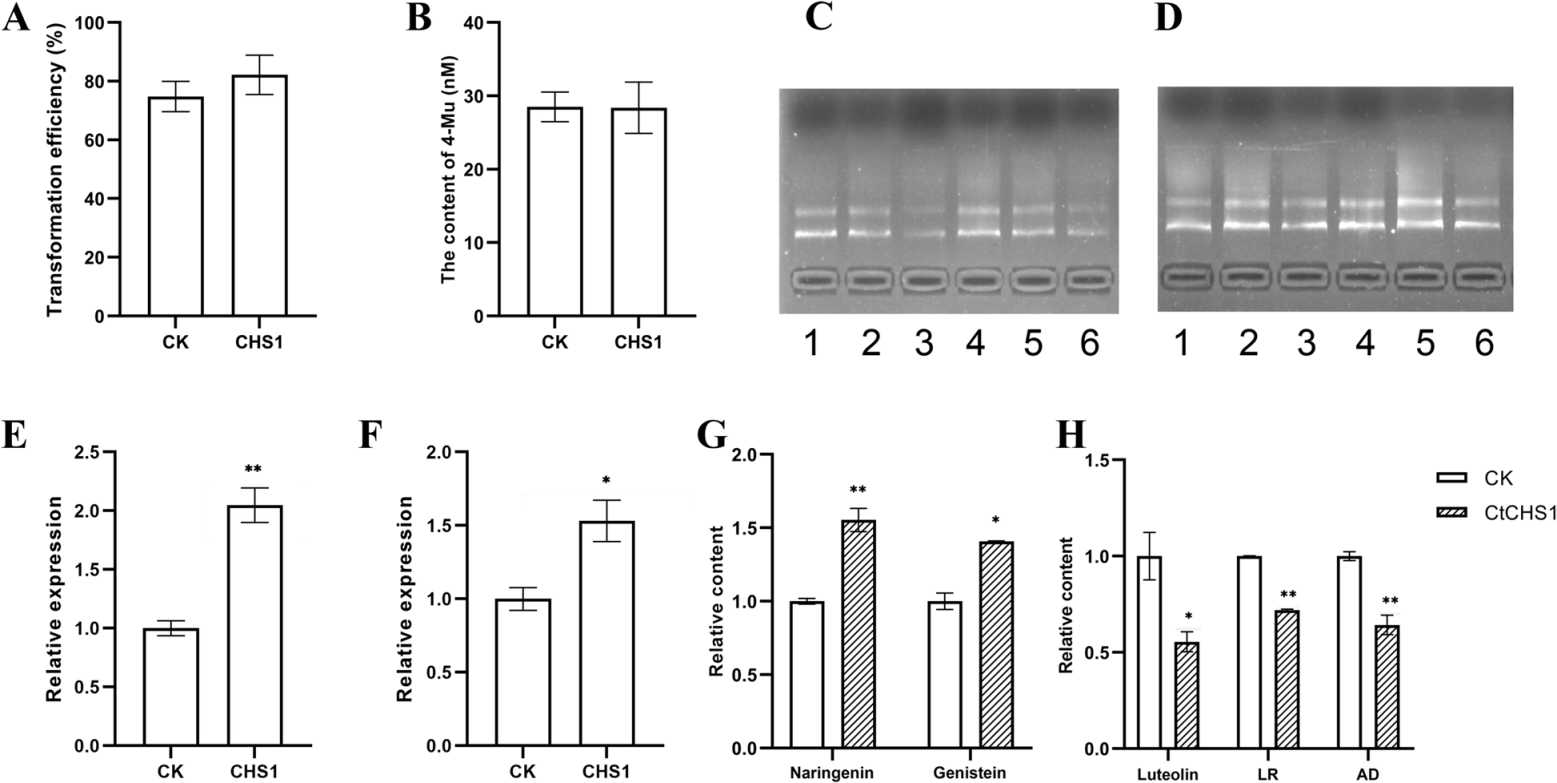
Results of *Agrobacterium* and biolistic-mediated overexpression of CtCHS1 in safflower callus. **A**, **B** The transform efficiency of *Agrobacterium* and biolistic-mediated overexpression. **C**, **D** The RNA extracted from *Agrobacterium* and biolistical transformed callus. **E**, **F** The relative expression of CtCHS1 in *Agrobacterium* and biolistic transformed and control callus. **G** Naringenin and genistein were significantly increased in *Agrobacterium*-transformed callus. H Luteolin, luteolin-7-O-rutinoside and apigenin derivative were significantly decreased in biolistic-transformed callus. The data are mean ± SEM (*n* = 3), * indicates statistical signifcance at *p* < 0.05 and ** indicates statistical signifcance at *p* < 0.01

After optimizing the experimental parameters of the two transient transformation systems, *CtCHS1* was overexpressed in calli to determine whether the developed transient expression systems could be used to investigate gene function in safflower. First, The *CtCHS1* gene was amplified from safflower’s cDNA, linked to the were cloned into a *pMD19-T* vector (Tiangen, China) and validated by sequencing. The results showed that the *CtCHS* gene was successfully cloned (Fig. 4A). Then, the expression vectors *pRI201-CtCHSI* and *pBI221-CtCHSI* were constructed (Fig. 4B, C). The *pRI201-CtCHS1* plasmid was finally transferred into the EHA105 *Agrobacterium* strain for infiltration. *pBI221-CtCHSI* was then amplified in *E.* coli, yielding a large number of plasmids for biolistic transformation. Finally, the two transient expression systems were used to transform *CtCHS1* into safflower calli for overexpression; empty vectors *pRI201* and *pBI221* were used as controls, respectively. Transformation efficiency was compared between the control and experimental groups (Fig. 5A, B), although no significant differences were noted, indicating that the obtained calli using the same transformation method possessed similar properties.

**Fig. 6:**
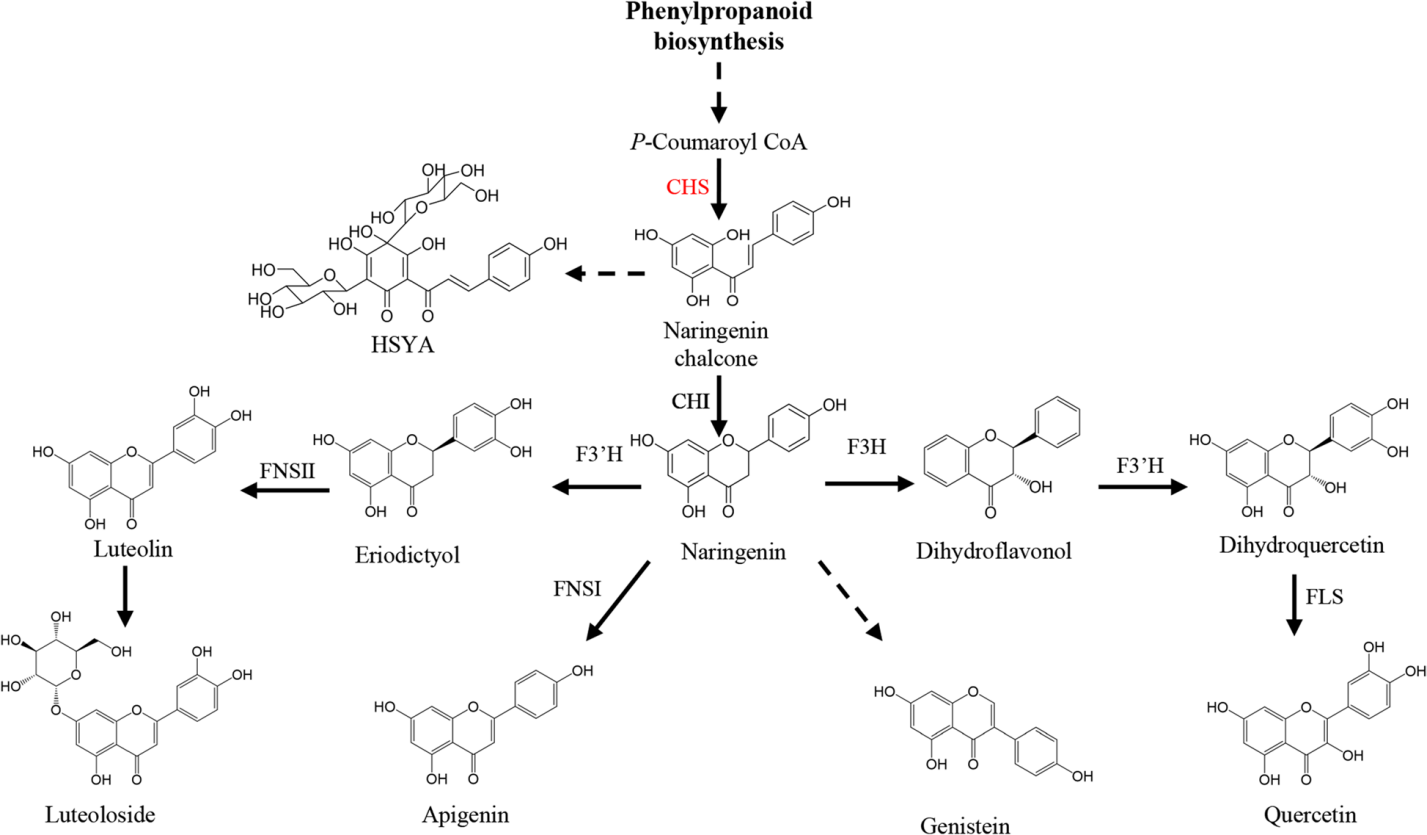
Biometabolic pathways of some flavonoid compounds in saffron. CHI, chalcone isomerase; CHS, chalcone synthase; HSYA, hydroxysafflor yellow A; FLS, flavonol synthase; FNSI, flavone synthase I; FNSII, flavone synthase II; F3H, flavanone 3-hydroxylase; F3’H, flavonoid 3’-hydroxylase

Furthermore, RNA (Fig. 5C, D) from the transformed groups and control groups were extracted and reverse-transcribed in cDNA for quantitative PCR analysis. Compared with that in the control calli, *CtCHS1* expression was significantly increased in the transformed calli (Fig. 5E, F), indicating that *CtCHS1* was successfully overexpressed in safflower calli using the developed *Agrobacterium*- and biolistic-mediated systems. Next, the total flavonoid content of calli was analyzed using LC–MS. Six flavonoids were detected: luteolin, luteolin-7-O-rutin (LR), naringenin, apigenin derivative (AD), genistein, and dihydroquercetin (Fig. 6). In *Agrobacterium*-transformed safflower calli, naringenin and genistein levels were significantly increased in the overexpression group (Fig. 5G). In biolistic-transformed safflower callus, luteolin, LR and AD levels were significantly decreased in the overexpression group (Fig. 5H).

## Discussion

Transient expression systems are efficient tools for investigating gene function, and these systems are advantageous in that they can be applied in some medicinal plants, such as *Artemisia annua* [16], *Salviae Miltiorrhizae* Radix et Rhizoma [17], and *Taraxacum officinale* [18]. Transient expression materials are diverse, including calli, protoplasts, leaves, flowers, and fruits, and each explant has its own features. As a member of the Asteraceae family, safflower’s features, such as achenes, leathery leaves, and minute tubular flowers, are not conducive to the direct application of transient transformation. Transient expression systems using protoplasts of safflower have been used for gene functional analysis [19]. However, the preparation of safflower protoplasts is affected by the flowering period and requires extensive experimentation. In this context, calli have the advantages of not being limited by seasons, having a stable genotype, and loose texture, allowing for easier production of more positive samples. Transient expression with calli has been used in many plants, such as *Mesembryanthemum crystallinum* [20], *Pinus tabulaeformis* [21], and *Sophora fragrans* [22]. Therefore, in the present study, calli were induced from the cotyledons of sterile safflower seedlings. Calli present a consistent genetic background and can achieve a high level of expression efficiency, which is suitable for transient expression.

*Agrobacterium* is commonly employed for transient expression; however, many factors can affect transformation efficiency. The *Agrobacterium* strain EHA105 shows high infectivity in various plants, such as *Gossypium hirsutum* [23], *Aloe barbadensis* [24], and *Artemisia annua* [16]. In the present study, the *Agrobacterium* strain EHA 105 was used to explore the effects of several factors, including the original *Agrobacterium* density, *Agrobacterium* concentration, infection time, co-cultivation time, and AS concentration. The transformation rate was the highest when the original *Agrobacterium* concentration was OD_600_ = 0.4, as the growth activity of *Agrobacterium* at this concentration was the highest. Among different *Agrobacterium* concentrations, the highest number of positive samples was noted at the OD_600_ value of 0.6. Low *Agrobacterium* concentration may lead to insufficient contact between *Agrobacterium* and calli, resulting in low transformation efficiency; however, excess concentration may lead to the browning or death of explants and decrease in the transformation rate. Furthermore, infection time affects the full contact between *Agrobacterium* and callus. However, a prolonged infection time may damage the callus. In our experiments, the highest conversion rate was reached after 3 days of co-cultivation, consistent with the experimental results of Song et al. [25]. Sufficient co-cultivation time is favorable for the transfer of T-DNA to callus; however, since the medium does not contain antibiotics, excessive culturing time leads to *Agrobacterium* overgrowth. Since AS activates the vir region of *Agrobacterium*, it improves the transformation efficiency of T-DNA. The sensitivities of different *Agrobacterium* strains and explants to AS vary [26]. For example, when the leaves of *Maesa lanceolata* were transformed, the transformation efficiency was the highest at the AS concentration of 100 μmol·L^−1^ [27]; in *Nicotiana benthamiana*, 500 μmol·L^−1^ AS achieved the best effect on tobacco leaves [28]. In the present study, the best effect was achieved when the AS concentration was 100 μmol·L^−1^. However, at concentrations exceeding this value, the transformation rate decreased, due perhaps to the toxic effects of AS on the explants.

Furthermore, biolistic is a commonly used transient expression method. During the transformation process, many parameters affect efficiency, such as helium pressure, bombardment distance, vacuum, bombardment times, gold particle amount, and plasmid amount [29]. Helium pressure determines the speed of gold particle, and sufficient speed helps penetrate the cell wall and deliver DNA. In the present experiments, a higher transformation efficiency was noted at 1,350 psi pressure, which is consistent with the experimental results in *Phoenix dactylifera* ‘Estamaran’ [30] and peanuts [31]. Similarly, vacuum can affect the speed of particles. The higher the degree of vacuum, the lower the power loss of particles during flight; however, if the degree of vacuum is very high, water loss and viability reduction occur in the explants, resulting in a lower transformation efficiency [32]. Furthermore, bombardment distance affects the final velocity and distribution of gold particles. The longer the bombardment distance, the slower the speed of the gold particles and the more discrete their distribution. In the present experiment, with increase in the bombardment distance, the expression of GUS in safflower calli decreased, which is consistent with reports by Narra et al. [33]. Increasing the number of bombardments increases the probability of plasmids entering the explant; however, there were no differences in GUS expression among safflower calli bombarded for different durations. These results are consistent with reports on *Tripterygium wilfordii* [34] and wheat [35]. Additionally, increasing the concentration of gold particles may decrease the expression of GUS [36]. This may be because a limited concentration of plasmid cannot adequately encapsulate the gold particles, and a high density of gold powder particles can damage the cell wall and membrane. In addition, excess gold particles can easily aggregate during processing, and the agglomerated gold particles may lead to the death of the recipient cells [37]. Within a certain range, expression efficiency may increase with increasing plasmid concentration; however, at excess plasmid concentration, gold particles may agglomerate, reducing the transformation efficiency.

Transient expression systems can be used for the analysis of gene functions [38, 39], protein interactions, and subcellular localization [40, 41]. For instance, after transient expression of *MdMYBPA1* in apple calli, significant increases in the content of proanthocyanidin and expression levels of several flavonoid synthetic genes were observed, demonstrating that *MdMYBPA1* activates proanthocyanidin synthesis in apples [42]. Although transient expression systems have been established for many plants reasons, whether these systems can in fact achieve their original purpose remains unclear. Therefore, after the initial establishment of systems, *CtCHS1* was overexpressed in safflower calli using the developed methods to verify their feasibility. After transient expression, transformation efficiency in the control and experimental groups was compared. Notable, no significant differences were noted in the transformation efficiency between the control and experimental groups of both systems, indicating that the experimental conditions were relatively consistent. Furthermore, qPCR analysis showed that the expression levels of *CtCHS1* in the experimental group were significantly higher than those in the control group, indicating that both transient expression systems successfully mediated gene overexpression. *CtCHS1* selected in the present study alters the contents of specific flavonoids in safflower. Accordingly, changes in the contents of certain flavonoid were indeed observed in calli transformed using the two methods. Specifically, the contents of naringenin and genistein were significantly increased in *Agrobacterium*-transformed safflower calli, whereas those of luteolin, LR, and AD were significantly decreased in biolistic-transformed safflower calli; therefore, both transient expression systems can be used for gene functional analyses. The *Agrobacterium* transient expression method has the advantage of high efficiency and the ability to transform a wide range of plants and tissues; however, it has the disadvantage of causing cell death, affecting plant growth and interfering with the expression of target genes. The biolistic transient expression method does not need to infect cells, and the introduction of DNA into plant cells by high-speed particle bombardment does not affect the growth of the recipient, making it suitable for almost all plants and tissues. However, compared with the *Agrobacterium* transient expression system, the biolistic transformation method is less efficient, consumes more DNA, has expensive equipment, and is more costly. Both *Agrobacterium* transient expression method and gene gun transient expression method are commonly used for exogenous gene expression, post-transcriptional gene silencing, promoter analysis, protein subcellular localization and interactions. Different plants may have different suitable transient expression methods. For example, *Arabidopsis thaliana* [8] and *Nicotiana benthamiana* are often transiently expressed using *Agrobacterium*, while rice [43] and maize [44] are often transiently expressed using gene guns. Moreover, these two transient expression methods could produce different results, and these differences are determined by a combination of expression vector, transformation efficiency, target gene, and experimental conditions [45]. Therefore, without knowing which system applies to safflower, we have established both transient expressions. Overall, both methods have their own advantages and disadvantages, and the choice of which method to use should be evaluated and selected based on the needs of the specific experiment. We have added the corresponding content to the discussion section.

## Conclusions

Safflower is a well-known traditional medicinal plant and its flavonoid metabolites have attracted much research attention. In recent years, the whole genome sequence of safflower has been published, and many genes related to flavonoid synthesis have been identified [46]. However, much work remains to fill in the knowledge gaps in gene function. The two transient expression systems established in the present study show high efficiency and can be applied for gene function analysis. Our callus-based experiments have built a foundation for subsequent transgenic research in safflower.

In this study, the transient expression system mediated by *Agrobacterium* and biolistic was successfully established, the optimal experimental conditions were screened. The two transient expression systems established in this experiment have the characteristics of high transformation efficiency, and can be practically used for gene function analyze. In the background of the publication of safflower whole genome data and the identification of many genes, the establishment of the transient expression system will lay a solid foundation for subsequent gene function identification studies.

## Materials and methods

### Plant material and treatments

Seeds were collected from *Carthamus tinctorius* cultivated in the medicinal botanical garden of the Chengdu University of Traditional Chinese Medicine, Chengdu City, Sichuan Province, China, and stored at 4 °C. The safflower seeds were rinsed under running water for 4 h, disinfected with 0.1% HgCl_2_ for 12 min, and rinsed three times with sterile water. The sterilized safflower seeds were sown in MS medium (Table 1) under 16/8 h light/dark conditions at 25 ± 2℃ [47]. After 7 days, sterile safflower seedlings were obtained.

**Table 1:** The composition of different media used in the study

Media	Composition
MS medium	1/2 MS, 30 g·L^−1^ source, 6 g·L^−1^ agar
Induction medium	MS, hormones, 30 g·L^−1^ source, 6 g·L^−1^ agar
Suspension solution	1/2 MS, 30 g·L^−1^ source
Co-cultivation medium	MS, 0.1 mg·L^−1^ NAA、2 mg·L^−1^ 6-BA、15 mg·L^−1^ KT-30, 30 g·L^−1^ source, 6 g·L^−1^ agar
Hypertonic medium	MS, 0.4 M·L^−1^ mannitol, 30 g·L^−1^ source, 6 g·L^−1^ agar

Callus was induced from the cotyledons of the sterile safflower seedlings. For safflower callus formation, six induction media containing different hormones were prepared (Table 2). In addition to the hormones, the media contained 4.43 g·L^−1^ MS salts, 30 g·L^−1^ sucrose, and 8 g·L^−1^ agar (Table 1). The medium pH was adjusted to 5.8–6.0, and it was sterilized at 121℃ for 20 min before being dispensed into Petri dishes. The margins and midribs of cotyledons were removed, and wounds were created on the backside; then, the explants were divided into 0.5 cm^2^ pieces and inoculated with the backside down in the prepared media. The inoculated cotyledons were cultured in the dark at 20℃ for 40 days.

**Table 2:** The hormone of safflower callus induction (mg·L^−1^)

NO	NAA	6-BA	KT-30
①	0.1	2	10
②	0.1	2	15
③	0.1	2	20
④	0.1	2	0
⑤	0.5	2	0
⑥	1.0	2	0

### Agrobacterium‑mediated transient transformation

The binary plasmid vector *pRI201* harboring the *GUS* and *CaMV 35S* promoters was selected for the experiments. The plasmids were introduced into competent *Agrobacterium tumefaciens* EHA105 (Tsingke, China) cells using the freeze–thaw method. The *Agrobacterium* suspension was spread on LB plates supplemented with 50 mg·L^−1^ kanamycin and 20 mg·L^−1^ rifampicin and cultured at 28℃ for 2–3 days in the dark.

A single colony of *Agrobacterium* was inoculated into LB liquid medium supplemented with the same antibiotics and incubated at 28℃ for 3–4 h at 180–220 rpm in the dark. The bacterial suspension was inoculated into several media containing antibiotics, and *Agrobacterium* cells were harvested when the density reached OD_600_ = 0.3, 0.4, 0.5, 0.6, and 0.7. *Agrobacterium* cells were collected by centrifuging at 5,000 rpm for 12 min, and then rinsed two times with the suspension (Table 1). Finally, the *Agrobacterium* cells were suspended to reach the required OD_600_.

The safflower callus was infiltrated with the *Agrobacterium* suspension, and the steps were as follows (text in parentheses indicates experimental parameters). (1) The safflower callus was placed into a sterile centrifuge tube, and the *Agrobacterium* suspension with the original concentration of OD_600_ = 0.6 (OD_600_ = 0.3, 0.4, 0.5, 0.6, or 0.7) and OD_600_ = 0.4 (OD_600_ = 0.2, 0.4, 0.6, 0.8, or 1.0) was added to the tube until the callus submerged. (2) The centrifuge tube was gently inverted to enable full contact between the callus and *Agrobacterium*. (3) After 15 min (5, 10, 15, 20, and 25 min), the *Agrobacterium* suspension was removed. (4) The callus was moved to a co-cultivation medium (Table 1) and cultured at 28℃ for 2 days (1, 2, 3, 4, and 5 days) in the dark. In experiments exploring the effect of AS concentration on the infection efficiency, AS was added at final concentration of 50, 100, 150, 200, or 250 mM·L^−1^.

The transformed calli were submerged in a GUS staining solution (Coolaber. China) for 24 h in the dark at 37℃. After removing the staining solution, the samples were washed three times with 75% ethanol. The calli with more than one-third stained area were considered positive. Three biological replicates were performed.

### Biolistic‑mediated transient transformation

The binary vector *pBI221* was used to optimize the parameters for biolistic-mediated transient transformation. The vector contained a *GUS* reporter gene and the *CaMV 35S* promoter. The plasmids were transformed into competent *Escherichia coli* DH5α (Tsingke, China) cells to obtain sufficient plasmids for the experiments. The transformed *E. coli* cells were cultured in the dark at 37 °C, and the plasmids were extracted.

Gold particles were prepared as previously described [48]. Briefly, (1) gold particles (1.0 μm, 50 mg; Bio-Rad) were washed two times each with 75% ethanol, followed by sterile water, and 50% sterile glycerol was added to achieve the final gold particle concentration of 50 mg·mL^−1^. (2) The gold particle solution was vortexed to distribute them evenly in the solution, and 4 μL of the solution was rapidly pipetted (when optimizing gold particle concentration, 2, 4, 6, and 8 μL of the solution were pipetted) into a sterile 1.5 mL centrifuge tube. (3) During vortexing, 2 μL of the plasmid suspension (1,000 ng·μL^−1^) (when optimizing plasmid concentration, 1, 2, 3, and 4 μL of the plasmid suspension was pipetted), 25 μL of 2.5 mol·L^−1^ CaCl_2_ solution, and 10 μL of 0.1 mol·L^−1^ spermidine solution were added to the centrifuge tube in that order. (4) The samples were centrifuged at 5,000 rpm for 5 min to collect the gold particles, which were washed two times with 200 μL of 75% and 100% ethanol. (5) The microparticles coated with plasmids were resuspended in 20 μL of 100% ethanol and temporarily placed on ice. Microcarriers prepared according to the above steps were used for bombardment.

A Bio-Rad 1000/He PDS particle delivery system (Bio-Rad, USA) was used, and all components were sterilized. The safflower calli were pre-cultured in a hypertonic medium (Table 1) for 4 h before bombardment. The calli were transformed at different conditions of gold particle concentration (100, 200, 300, and 400 μg·shot^−1^), plasmid concentration (1, 2, 3, and 4 μg·shot^−1^), helium pressure (1,100 and 1,350 psi), vacuum (-0.2, -0.4, -0.6, -0.8, and -1.0 bar), flight distance (6.5, 8.0, 9.5, and 11.0 cm), and bombardment rounds (one, two, and three times). The transformed calli were cultured in the dark at 28℃ in a hypertonic medium for 24 h.

The callus transformation efficiency was represented in terms of the enzymatic activity of GUS. Successfully transformed calli produce GUS protease, which catalyzes the conversion of 4-methylumbelliferone glucuronide (4-MUG) into fluorescent 4-methylumbelliferone (4-MU). By measuring fluorescence, GUS, can be quantitatively detected. Transformation efficiencies under different experimental conditions were determined according to the kit protocol (Coolaber, China), and three biological replicates were performed.

### Agrobacterium- and biolistic-mediated CtCHS1 overexpression in safflower calli

Safflower flowers were collected from the medical botanical garden of the Chengdu University of Traditional Chinese Medicine. RNA was extracted from flowers and reverse-transcribed into cDNA. The *CtCHS1* sequence was derived from a previous study, which showed that *CtCHS1* significantly affects the accumulation of certain flavonoids [15]. The specific primers *CtCHS1-F* and *CtCHS1-R* (Table 3) were designed using Primer Premier 5.0. PCR was performed as follows: initial denaturation at 98℃ for 3 min; 34 cycles of 98℃ for 30 s, 56℃ for 30 s, and 72℃ for 2 min; and final extension at 72℃ for 8 min. PCR products were cloned into a *pMD19-T* vector (Tiangen, China) for sequencing (Tsingke, China).

**Table 3:** Primers for gene cloning and vector construction

Primer	Sequence
CtCHS1-F	ATGGCATCCTTAACCGATATTG
CtCHS1-R	TTAAGCGGCAATGGGGGTGG
pRI201-CHS1-F	TCTACAGGACGTAACATATGATGGCATCCTTAACCGATAT
pRI201-CHS1-R	CTTCACTGTTGATACATATGTTAAGCGGCAATGGGGGTGG
pBI221-CHS1-F	TGTTGATAGTCGACGGATCCATGGCATCCTTAACCGATAT
pBI221-CHS1-R	ACCACCCGGGGATCCTTAAGCGGCAATGGG
qCtCHS1-F	CTGCCACAAAAGCCATTA
qCtCHS1-F	ACGGAAGGTGACCGCGGTGATCTCG
25S-F	GGAGGTTGAGGGAAAAGGAG
25S-R	GTGACCTCGTCACCCGTAGT

The *pRI201* plasmid was used for *Agrobacterium*-mediated transient expression, and the restriction enzyme site was NdeI (Takara, Japan). The *pBI221* plasmid was used for biolistic-mediated transient expression, and the restriction enzyme site was BamHI (Takara, Japan). Gene-specific primers were designed based on the open reading frame (ORF) of *CtCHS1* and restriction enzyme sites (Table 2). The *CtCHS1* fragments were cloned into *pRI201* and *pBI221*, and the plasmids were verified by sequencing (Tsingke, China).

The constructed *pRI201-CtCHS1* plasmid was extracted from *E. coli*, transformed into EHA105 *Agrobacterium*-competent cells, and introduced into safflower calli under the optimized experimental conditions. After multiplication in *E. coli*, the constructed p*RI201-CtCHS1* plasmid was extracted, loaded onto gold particles, and delivered into safflower calli under optimized conditions.

Transformed safflower calli were divided into three groups. One was used to measure transformation efficiency. One was used to extract RNA, which was reverse-transcribed into cDNA for quantitative real-time PCR (q-PCR) (Bio-Red, USA). q-PCR was used to detect *CtCHS1* expression levels. The 25S rRNA gene of *Carthamus tinctorius* was used as the internal reference. Specific primers were designed using Primer Premier v5. q-PCR was performed as follows: 95 °C for 3 min, followed by 40 cycles of 95℃ for 10 s and 58.5℃ for 30 s [49]. The remaining samples were freeze-dried and ultrasonically extracted two times in water to obtain total flavonoids. The extracts were dissolved in methanol and analyzed using LC–MS (Synapt, Waters, USA). The experimental steps are described elsewhere [19]. Safflower calli transformed with an empty vector were used as controls. Three biological replicates were performed.

### Statistical analysis

Statistical analyses of the callus induction rate, GUS straining frequency of *Agrobacterium*-transformed calli, GUS enzymatic activity in biolistic-transformed calli, *CtCHS1* gene expression level, and relative flavonoid content were performed using Student’s *t*-test (*p* < 0.01). The callus induction rate (%) (number of successfully induced calli/total number of unstained explants × 100%), *Agrobacterium*-transformation efficiency (%) (number of positive calli/total number of infected calli × 100%), GUS enzymatic activity (calculated from established standard curve) after biolistic-transformation, and relative flavonoid content (peak area of experimental group/peak area of control group) were calculated. Each experiment was independently repeated three times.

## Supplementary Information

**Additional file 1:** 

## Data Availability

All data generated or analyzed during this study are included in this published article.

## Abbreviations

AD: Apigenin derivative
AS: Acetosyringone
CHI: Chalcone isomerase
*CHS1*: Chalcone synthase
CP1: Cysteine protease 1
CYP: Cytochrome P450
FLS: Flavonol synthase
FNSI: Flavone synthase I
FNSII: Flavone synthase II
F3H: Flavanone 3-hydroxylase
F3’H: Flavonoid 3’-hydroxylase
HSYA: Hydroxysafflor yellow A
GUS: β-Glucuronidase
LR: Luteolin-7-O-rutinoside
T-DNA: Transfer DNA
4-MU: Methylumbelliferone
