# Supplementary material for: The establishment of transient expression systems and their application for gene function analysis of flavonoid biosynthesis in Carthamus tinctorius L
Source: BMC Plant Biol. 2023 Apr 10;23:186. doi: 10.1186/s12870-023-04210-1 (PMC10084634; doi:10.1186/s12870-023-04210-1)
Supplement: Supplementary file 1 — Additional file 1: [file 12870_2023_4210_MOESM1_ESM.pdf]

Supplementary Info File

The complete picture of gel electrophoresis images in Fig. 4 (The part in the red box is the part shown in Fig. 4).

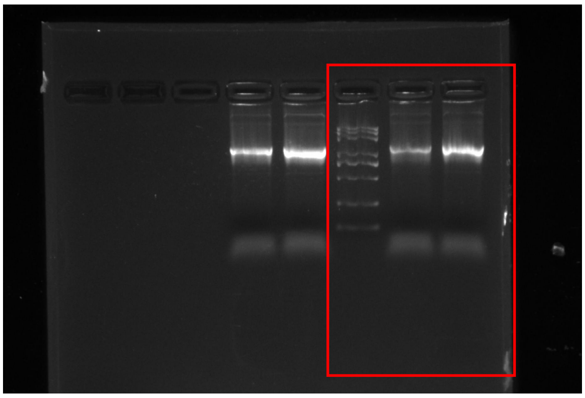

Fig. 4A

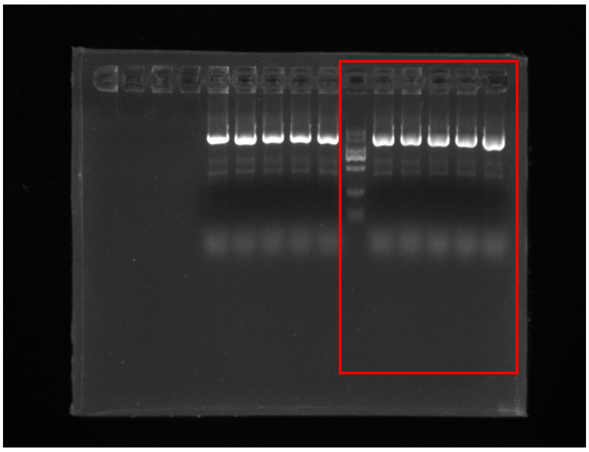

Fig. 4B

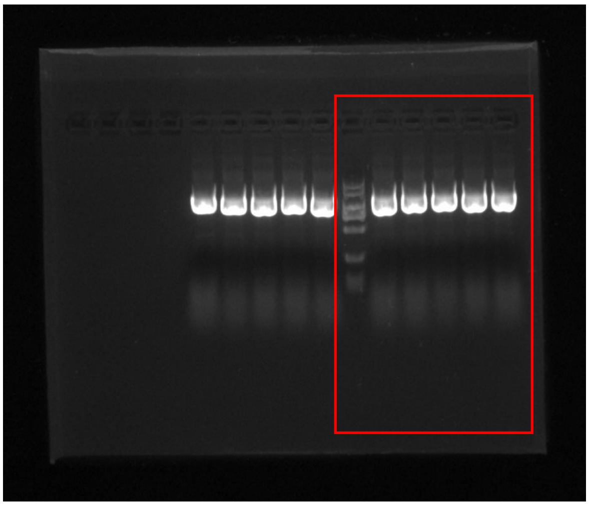

Fig. 4C
